# Improved genome assembly of *Aspergillus niger* ATCC 1015, a citric acid-producing isolate

**DOI:** 10.1128/mra.00281-26

**Published:** 2026-05-29

**Authors:** Adéla Schandl, Julien Charest, Astrid R. Mach-Aigner, Matthias G. Steiger

**Affiliations:** 1 Christian Doppler Laboratory for Sustainable Bioproduction with Fungal Systems through Targeted Strain Development, Institute of Chemical, Environmental and Bioscience Engineering, Research Group Biochemistry, Technische Universität Wien 210802 , Vienna, Austria; 2 Institute of Chemical, Environmental and Bioscience Engineering, TU Wien 210802 , Vienna, Austria; University of Maryland, Baltimore, Maryland, USA

**Keywords:** citric acid, Illumina, Oxford Nanopore, mitochondrial genome, organic acids

## Abstract

We present a high-quality hybrid genome assembly of *Aspergillus niger* ATCC 1015, a citric acid-producing isolate. The genome consists of eight nuclear chromosomes with a length of 35,617,925 bp (N50: 4.3 Mb; GC: 49.6%) and a 31,333 bp mitochondrial genome, providing a valuable resource for biotechnological research and applications.

## ANNOUNCEMENT

*Aspergillus niger* ATCC 1015 is an industrial strain used for citric acid production (1). Here, we present a new hybrid Nanopore-Illumina genome assembly of this strain, improving contiguity over the prior versions (2, 3). This resource enhances the genetic foundation and supports strain engineering for biotechnological applications.

*Aspergillus niger* ATCC 1015, obtained from the Westerdijk Fungal Biodiversity Institute (CBS 113.46) in April 2025, was passaged twice prior to sequencing on minimal medium (1.5% agar, 1% glucose, 1× ASPA + N, 0.002 M MgSO₄, and 1× trace elements solution (4)). Conidia were inoculated into complete medium supplemented with 0.5 g/L casein peptone, 2.5 g/L yeast extract, 1% glucose, 1× ASPA + N, 0.002 M MgSO₄, and 1× trace elements solution (4) and incubated for 16 h at 30°C with shaking at 150 rpm. The mycelium was harvested, and protoplasts were prepared using 40 mg/mL VinoTaste (Novozymes, Bagsværd, Denmark) lysing enzymes in a buffer containing sorbitol, MES, and CaCl_2_ (4), incubated at 37°C for 2.5 h. Protoplasts were resuspended in Zymo DNA/RNA Shield (Zymo Research, Irvine, USA).

Protoplasts of *Aspergillus niger* ATCC 1015 were sent to Plasmidosaurus for DNA extraction and custom sequencing using Oxford Nanopore Technology (ONT) and Illumina platform. Genomic DNA was isolated using the Monarch Genomic DNA Purification Kit (New England Biolabs, USA) without modifications and without shearing or size selection. Nanopore libraries (v14 chemistry; tagmentation) were prepared using the SQK-RBK114.96 kit (Oxford Nanopore Technologies, UK) and sequenced on a PromethION 24 using two R10.4.1 flow cells. Basecalling was performed with Guppy v6.4.6 (Oxford Nanopore Technologies; super-accurate mode, Q ≥ 10), and adapters and barcodes were trimmed using MinKNOW (Oxford Nanopore Technologies), yielding 521,857 reads in total (243,482 reads, N50 9,597 bp; 278,375 reads, N50 9,419 bp). Illumina libraries were prepared using the Illumina DNA Prep kit (Illumina, USA) and sequenced on a NextSeq 2000 platform as paired-end reads (2 × 150 bp), generating 28.5 million read pairs. Reads were first decontaminated against the human reference genome GRCh38.p13 (5): ONT using minimap2 v2.30 (6) and Illumina using bwa v0.7.19 (7). Unmapped ONT reads and Illumina read pairs were then extracted with samtools v1.22.1 (8). Finally, ONT reads were deduplicated (seqkit v2.10.1 (9)), quality-filtered (filtlong v0.3.0 (https://github.com/rrwick/Filtlong); top 95%, ≥3 kb), and Illumina reads were adapter-trimmed and quality- and length-filtered with fastp v1.0.1 (10) (Q ≥ 20, ≥50 bp).

The hybrid genome of *A. niger* ATCC 1015 was assembled *de novo* with Flye v2.9.6 in the nano-hq mode with an estimated genome size of 36 Mb (scaffolded) using processed Nanopore reads and polished with Illumina reads via Pilon v1.24 (11). Assembly quality was assessed using QUAST v5.3.0 (12) and BUSCO v5.7.1 (13) with the ascomycota_odb10 lineage data set, showing 99.6% BUSCO completeness. The assembly consists of nine contigs (including the mt-genome), with the nuclear genome composed of eight chromosomes at 35,617,925 bp (N50 4,304,314 bp; GC 49.59%) and one mtDNA at 31,333 bp. All chromosome-scale contigs terminate in tandem arrays with the motif TTTTAGGG, an elongated variant of the canonical telomeric repeat TTAGGG, which is also found in algae such as *Chlamydomonas reinhardtii* (14). Sequencing coverage was 59× for Nanopore reads and 197× for Illumina reads.

The mitochondrial contig was annotated using a reference-guided, manually curated approach, combining exonerate v2.4.0 (protein2genome model against *Aspergillus flavus* proteins [15]), tRNAscan-SE v2.0.12 (16) for tRNAs, and Infernal cmscan (Rfam) v1.1.5 for rRNAs; features were merged using AGAT v1.5.1 (17). The nuclear genome was annotated using funannotate v1.8.17 (18), including repeat masking, gene prediction with Augustus (19) (*Aspergillus nidulans* model), and functional annotation assigned by eggNOG-mapper v2.1.13 (20), resulting in 11,387 protein-coding genes. Liftoff v1.6.3 (21) was used to transfer features from the Aspni5 assembly (2), and the annotations were then merged using gffcompare v0.12.10 (22). During this process, protein ID-derived aliases were added, and hypothetical mRNAs were replaced with more informative gene names in the final GFF3 file.

## Data Availability

Raw sequencing reads have been deposited in the Sequence Read Archive (SRA) under BioProject PRJNA1401865. Illumina short-read data are available under SRA run accession number SRR36864153. ONT long-read data are available under SRA run accession number SRR36864154. The nuclear whole-genome shotgun (WGS) project for *Aspergillus niger* ATCC 1015, associated with BioSample SAMN54575164, has been deposited in DDBJ/ENA/GenBank under the accession number JBTSWI000000000. The version described in this paper is the first version, JBTSWI010000000. The mitochondrial genome sequence has been deposited in DDBJ/ENA/GenBank under the accession number PX959665.

## References

[1] Karaffa L, Kubicek CP. 2003. Aspergillus niger citric acid accumulation: do we understand this well working black box? Appl Microbiol Biotechnol 61:189–196. doi:10.1007/s00253-002-1201-7 12698275

[2] Andersen MR, Salazar MP, Schaap PJ, van de Vondervoort PJI, Culley D, Thykaer J, Frisvad JC, Nielsen KF, Albang R, Albermann K, et al.. 2011. Comparative genomics of citric-acid-producing Aspergillus niger ATCC 1015 versus enzyme-producing CBS 513.88. Genome Res 21:885–897. doi:10.1101/gr.112169.110 21543515 PMC3106321

[3] Ellena V, Steiger MG. 2022. The importance of complete and high-quality genome sequences in Aspergillus niger research. Front Fungal Biol 3:935993. doi:10.3389/ffunb.2022.935993 37746178 PMC10512394

[4] Arentshorst M, Ram AFJ, Meyer V. 2012. Using non-homologous end-joining-deficient strains for functional gene analyses in filamentous fungi, p 133–150. *In* Bolton MD, Thomma BPHJ (ed), Plant fungal pathogens: methods and protocols. Humana Press, Totowa, NJ. 10.1007/978-1-61779-501-5_9 22183652

[5] Schneider VA, Graves-Lindsay T, Howe K, Bouk N, Chen H-C, Kitts PA, Murphy TD, Pruitt KD, Thibaud-Nissen F, Albracht D, et al.. 2017. Evaluation of GRCh38 and de novo haploid genome assemblies demonstrates the enduring quality of the reference assembly. Genome Res 27:849–864. doi:10.1101/gr.213611.116 28396521 PMC5411779

[6] Li H. 2018. Minimap2: pairwise alignment for nucleotide sequences. Bioinformatics 34:3094–3100. doi:10.1093/bioinformatics/bty191 29750242 PMC6137996

[7] Li H. 2013. Aligning sequence reads, clone sequences and assembly contigs with BWA-MEM. arXiv:1303.3997. doi:10.48550/arXiv.1303.3997

[8] Danecek P, Bonfield JK, Liddle J, Marshall J, Ohan V, Pollard MO, Whitwham A, Keane T, McCarthy SA, Davies RM, Li H. 2021. Twelve years of SAMtools and BCFtools. Gigascience 10:giab008. doi:10.1093/gigascience/giab008 33590861 PMC7931819

[9] Shen W, Sipos B, Zhao L. 2024. SeqKit2: a Swiss army knife for sequence and alignment processing. iMeta 3:e191. doi:10.1002/imt2.191 38898985 PMC11183193

[10] Chen S. 2025. Fastp 1.0: an ultra‐fast all‐round tool for FASTQ data quality control and preprocessing. iMeta 4:e70078. doi:10.1002/imt2.70078 41112039 PMC12527978

[11] Walker BJ, Abeel T, Shea T, Priest M, Abouelliel A, Sakthikumar S, Cuomo CA, Zeng Q, Wortman J, Young SK, Earl AM. 2014. Pilon: an integrated tool for comprehensive microbial variant detection and genome assembly improvement. PLoS One 9:e112963. doi:10.1371/journal.pone.0112963 25409509 PMC4237348

[12] Gurevich A, Saveliev V, Vyahhi N, Tesler G. 2013. QUAST: quality assessment tool for genome assemblies. Bioinformatics 29:1072–1075. doi:10.1093/bioinformatics/btt086 23422339 PMC3624806

[13] Simão FA, Waterhouse RM, Ioannidis P, Kriventseva EV, Zdobnov EM. 2015. BUSCO: assessing genome assembly and annotation completeness with single-copy orthologs. Bioinformatics 31:3210–3212. doi:10.1093/bioinformatics/btv351 26059717

[14] Petracek ME, Lefebvre PA, Silflow CD, Berman J. 1990. Chlamydomonas telomere sequences are A+T-rich but contain three consecutive G-C base pairs. Proc Natl Acad Sci USA 87:8222–8226. doi:10.1073/pnas.87.21.8222 2236035 PMC54927

[15] Drott MT, Satterlee TR, Skerker JM, Pfannenstiel BT, Glass NL, Keller NP, Milgroom MG. 2020. The frequency of sex: population genomics reveals differences in recombination and population structure of the aflatoxin-producing fungus Aspergillus flavus. mBio 11:e00963-20. doi:10.1128/mBio.00963-20 32665272 PMC7360929

[16] Lowe TM, Eddy SR. 1997. tRNAscan-SE: a program for improved detection of transfer RNA genes in genomic sequence. Nucleic Acids Res 25:955–964. doi:10.1093/nar/25.5.955 9023104 PMC146525

[17] Jacques D. 2019. NBISweden/AGAT: AGAT-v0.0.1. Zenodo.

[18] Palmer JM, Stajich J. 2020. Funannotate v1.8.1: eukaryotic genome annotation. Zenodo.

[19] Stanke M, Diekhans M, Baertsch R, Haussler D. 2008. Using native and syntenically mapped cDNA alignments to improve de novo gene finding. Bioinformatics 24:637–644. doi:10.1093/bioinformatics/btn013 18218656

[20] Cantalapiedra CP, Hernández-Plaza A, Letunic I, Bork P, Huerta-Cepas J. 2021. eggNOG-mapper v2: functional annotation, orthology assignments, and domain prediction at the metagenomic scale. Mol Biol Evol 38:5825–5829. doi:10.1093/molbev/msab293 34597405 PMC8662613

[21] Shumate A, Salzberg SL. 2021. Liftoff: accurate mapping of gene annotations. Bioinformatics 37:1639–1643. doi:10.1093/bioinformatics/btaa1016 33320174 PMC8289374

[22] Pertea G, Pertea M. 2020. GFF utilities: GffRead and GffCompare. F1000Res 9:ISCB Comm J-304. doi:10.12688/f1000research.23297.2 PMC7222033 32489650

